# A histomorphometric meta-analysis of sinus elevation with various grafting materials

**DOI:** 10.1186/1746-160X-5-12

**Published:** 2009-06-11

**Authors:** Jörg Handschel, Melani Simonowska, Christian Naujoks, Rita A Depprich, Michelle A Ommerborn, Ulrich Meyer, Norbert R Kübler

**Affiliations:** 1 Department for Cranio- and Maxillofacial Surgery, Heinrich-Heine-Universität, Moorenstr. 5, D-40225 Düsseldorf, Germany; 2 Department for Operative and Preventive Dentistry and Endodontics, Heinrich-Heine-University Düsseldorf, Moorenstr. 5, D-40225 Düsseldorf, Germany

## Abstract

Several grafting materials have been used in sinus augmentation procedures including autogenous bone, demineralized freeze-dried bone (DFDBA), hydroxyapatite, β-tricalcium phosphate (β-TCP), anorganic deproteinized bovine bone and combination of these and others. Up to now a subject of controversy in maxillofacial surgery and dentistry is, what is the most appropriate graft material for sinus floor augmentation.

The aim of this study is to provide a body of evidence-based data regarding grafting materials in external sinus floor elevation concerning the fate of the augmented material at the histomorphological level, through a meta-analysis of the available literature.

The literature searches were performed using the National Library of Medicine. The search covered all English and German literature from 1995 until 2006. For analyzing the amount of bone the parameter "Total Bone Volume" (TBV) was assessed. TBV is determined as the percentage of the section consisting of bone tissue.

In a relatively early phase after implantation the autogenous bone shows the highest TBV values. Interestingly, the different TBV levels approximate during the time. After 9 months no statistically significant differences can be detected between the various grafting materials.

From a clinical point of view, the use of autogenous bone is advantageous if a prosthetic rehabilitation (with functional loading) is expected within 9 months. In other cases the use of anorganic deproteinized bovine bone in combination with autogenous bone seems to be preferable. Donor side morbidity is ignored in this conclusion.

## Introduction

Since the external sinus floor elevation technique was first introduced by Boyne [1] and Tatum [2] several grafting materials have been used in sinus augmentation procedures including autogenous bone [1-3], demineralized freeze-dried bone (DFDBA)[4,5], hydroxyapatite [6], β-tricalcium phosphate (β-TCP) [7], anorganic deproteinized bovine bone [8] and combination of these and others [9]. Up to now a subject of controversy in maxillofacial surgery and dentistry exist, what is the most appropriate graft material for sinus floor augmentation. The consensus conference on sinus grafting held in 1996 showed that in the light of little data which are evidence-based many participants believed that autografts were the most efficacious [10]. However, the collection of autogeneous bone requires an extra donor site surgery and carries with it extra risks for morbidity and complaints, particularly when bone from the iliac crest is harvested [11]. According to Kent and Block [3] an ideal grafting material should fulfil the following criteria amongst other things:

Osteoinduction

Osteoconduction

Volume stability

These criteria are best analysed by histological examinations. To the best of our knowledge, only a very small number of randomized controlled clinical trials have been conducted to compare various grafting materials with regard to these histological criteria. The available evidence therefore consists either of case reports, case series or retrospective studies. The aim of this study is to provide a body of evidence-based data regarding grafting materials in external sinus floor elevation to assist surgeons to make an informed choice between those materials, through a meta-analysis of the available literature.

## Methods

The literature searches were performed using the National Library of Medicine (Internet: ). The search covered all English and German literature from 1995 until 2006. Keywords used in the search were: "sinus" and "augmentation" and "bone substitute". The search was confined to studies or reports in humans. No animal studies were included. Moreover, review articles and in vitro studies were excluded. In all, 120 articles were identified and all abstracts were evaluated. After first evaluation the following inclusion criteria were added: The surgical procedure has to be an external sinus floor elevation and because of the presence of only single reports of some grafting materials – which does not allow a meta-analysis for those materials- the focus was on materials which are used in several studies/reports. Thus only papers using autogenous bone, demineralized freeze-dried bone (DFDBA), hydroxyapatite, β-tricalcium phosphate (β-TCP), anorganic deproteinized bovine bone (Bio Oss^®^, Geistlich Biomaterials, Wolhusen, Switzerland) [8] and combination of these materials were included. To standardize the multiple combinations of Bio Oss^® ^with autogenous bone all mixing ratios higher than 80% Bio Oss^® ^to 20% bone were pooled in the Bio Oss^® ^group. Mixing ratios below (e.g. 50% Bio Oss^® ^to 50% bone) were subsumed under the Bio Oss^® ^+ autogeneous bone group. Regarding the β-TCP group in almost all studies β-TCP was used without autogenous bone. In addition to review articles, interviews and editorials were excluded.

For analyzing the amount of bone the parameter "Total Bone Volume" (TBV) was assessed. TBV is determined as the percentage of the section consisting of bone tissue [12]. This parameter was either directly taken from the paper or calculated where possible. In studies reporting woven and lamellar bone separately, the sum of both values was calculated whereas in studies determing lateral and central bone biopsies the mean was calculated.

For statistical analysis the data were weighted according to the number of observations in each study and the inverse variance. Moreover, to detect any statistical significant differences a weighted ANOVA with Random effect model and alpha-adjustment according to Tukey-Kramer for post hoc tests was used [13]. Differences were considered statistically significant if p < 0.05.

## Results

After the initial literature search 120 articles were identified. Four of these articles were not written in English or german and another four were animal studies. Six articles were interviews or editorials and were excluded too. Of the remaining 106 articles 25 were not related to the external sinus floor elevation and another 16 articles gave an account on rare grafting material. Of the remaining 65 articles only in 30 studies the histomorphological parameter TBV was evaluable. That means that this parameter was explicitly noted in the article or could easily been calculated. Finally, only 30 articles remain for data analysis (table 1).

**Table 1:** Selection of evaluable articles by in- and exclusion criteria

**Criteria**	**Studies which do not meet the criteria**	**Remaining studies**
After initial literature search		120
English or german	4	116

Only human (no animal studies)	4	112

No interviews/editorials	6	106

Only external sinus floor elevation	25	81

Only autogenous bone, demineralized freeze-dried bone (DFDBA), hydroxyapatite, β-tricalcium phosphate (β-TCP), anorganic deproteinized bovine bone [8] and combination of these materials	16	65

TBV evaluable	35	30

In many of these 30 articles various grafting materials were described. In total 53 observations regarding grafting materials could be found. The vast majority were prospective studies, followed by some case reports or case series and finally one retrospective study (table 2).

**Table 2:** Distribution of articles and material observations

	**Σ**	**Case reports**	**Retrospective studies**	**Prospective studies**
Studies	30	3	1	26

Examined grafting materials (in these studies)	53	4	3	46

A prerequisite for statistical analysis is that the mean values and the standard deviation is noted in the paper (criteria I) [13]. This is not the case in single case reports (criteria II). That is why those papers meeting one of these two criteria have to be excluded from further analysis (table 3). If a meta-analysis for one specific grafting material would be based on only one or two studies, the result would almost echo the findings of the single study. Therefore, it is rational to exclude materials with only one or two reports (criteria III). Table 3 shows the number of remaining studies/observations after application of these three criteria (Tab. 3). Finally, 30 articles remain for evaluation [7,8,12,14-41]. The studies are listed in table 4 (table 4). In no studies any allergic reactions to grafting materials or infections related to graft implantation were described.

**Table 3:** Selection of evaluable material observations by three exclusion criteria

	**Total observation**	**Criteria I: no mean value or SD**	**Criteria II: single case report**	**Criteria I or II**	**Remaining material observations**
Algipore^®^	1	0	0	0	1

Bio Oss^®^	18	4	3	5	13*

Bio Oss^® ^+ autogen (50:50 bis 80:20)	8	0	0	0	8*

DFDBA	1	1	0	1	0

HA	3	1	0	1	2

Autogen	13	3	5	4	9*

Autogen + DFDBA 50:50	1	0	0	0	1

Autogen + HA 50:50	1	0	0	0	1

β-TCP	7	1	1	1	6*

Σ	53	17	5	18	41 (36)

Note the numbers with * show the grafting materials with at least three reported observations (criteria III).

**Table 4:** List of reviewed publications.

**Authors**	**Year of publication**	**Grafting material**	**Mean healing time (months)**	**n**	**TBV (%)**	**SD**
Artzi Z. et al.	2001	BioOss	12.00	10	32.20	8.150

Artzi Z. et al.	2001	HA	12.00	10	42.10	10.010

Artzi Z. et al.	2002	BioOss	12.00	10	43.61	8.601

Artzi Z. et al.	2003	HA	12.00	10	32.95	7.991

Artzi Z. et al.	2005	BioOss	12.00	12	45.60	10.900

Artzi Z. et al.	2005	β-TCP	12.00	12	32.00	8.400

Boeck-Neto RJ. et al.	2002	autogen+DFDBA	10.00	5	50.46	16.290

Boeck-Neto RJ. et al.	2002	autogen+HA	10.00	5	46.79	8.560

Degidi M. et al.	2004	BioOss+autogen	6.00	12	38.80	3.200

Froum SJ. et al.	2002	BioOss	7.25	2	16.00	4.243

Froum SJ. et al.	2002	BioOss	11.00	1	32.00	.

Fugazotto PA. et al.	2003	BioOss	6.88	26	52.85	19.605

Fugazotto PA. et al.	2003	BioOss	12.50	5	68.80	7.400

Hallman M. et al.	2001	BioOss+autogen	7.00	16	24.70	16.901

Hallman M. et al.	2001	BioOss+autogen	30.00	12	50.70	22.800

Hallman M. et al.	2002	Autogen	12.50	11	37.70	31.300

Hallman M. et al.	2002	BioOss	14.75	10	39.90	8.000

Hallman M. et al.	2002	BioOss+autogen	12.50	11	41.70	26.600

John HD. et al.	2004	Autogen	5.50	4	53.50	2.520

John HD. et al.	2004	BioOss	5.50	21	29.52	7.430

John HD. et al.	2004	BioOss+autogen	5.50	13	32.23	6.860

Karabuda C. et al.	2001	BioOss	6.00	5	50.00	.

Karabuda C. et al.	2001	DFDBA	6.00	1	72.50	.

Karabuda C. et al.	2001	HA	6.00	3	27.50	8.660

Ozyuvaci H. et al.	2003	BioOss	7.00	44	47.50	0.945

Ozyuvaci H. et al.	2003	β-TCP	7.00	44	52.50	0.945

Proussaefs P. et al.	2003	BioOss	11.00	5	34.40	10.810

Scarano A. et al.	2004	BioOss	53.00	1	38.00	.

Schopper C. et al.	2003	Algipore	7.00	26	23.00	8.300

Szabo G. et al.	2001	Autogen	6.00	4	37.05	8.842

Szabo G. et al.	2001	β-TCP	6.00	4	29.37	10.568

Szabo G. et al.	2005	Autogen	6.00	20	38.34	7.400

Szabo G. et al.	2005	β-TCP	6.00	20	36.47	6.900

Tadjoedin ES et al.	2000	Autogen	5.00	9	42.28	3.251

Tadjoedin ES et al.	2000	Autogen	16.00	1	45.07	.

Tadjoedin ES et al.	2002	Autogen	5.00	2	40.05	1.061

Tadjoedin ES et al.	2002	Autogen	15.00	1	41.70	.

Tadjoedin ES et al.	2003	Autogen	5.00	1	37.30	.

Tadjoedin ES et al.	2003	BioOss	8.00	1	22.90	.

Tadjoedin ES et al.	2003	BioOss+autogen	6.33	3	29.57	4.508

Trisi P. et al.	2003	BioOss+autogen	15.33	9	44.38	8.575

Turunen T. et al.	2004	Autogen	6.75	14	25.10	7.200

Turunen T. et al.	2004	Autogen	13.75	4	25.10	6.300

Valentini P. et al.	2000	BioOss	6.00	3	21.08	7.250

Valentini P. et al.	2000	BioOss	12.00	3	27.55	4.880

Wallace SS. et al.	2005	BioOss	8.00	153	15.53	8.023

Yildrim M. et al.	2000	BioOss	7.00	11	14.70	5.000

Yildrim M. et al.	2001	BioOss+autogen	7.75	12	18.90	6.400

Zerbo IR. et al.	2001	β-TCP	8.00	1	20.00	.

Zerbo IR. et al.	2004	Autogen	6.00	5	41.00	10.000

Zerbo IR. et al.	2004	β-TCP	6.00	9	17.00	5.000

Zijderveld SA. et al.	2005	Autogen	6.00	5	41.00	10.000

Zijderveld SA. et al.	2005	β-TCP	6.00	9	17.00	5.000

n = number of patients

**Figure 1:**
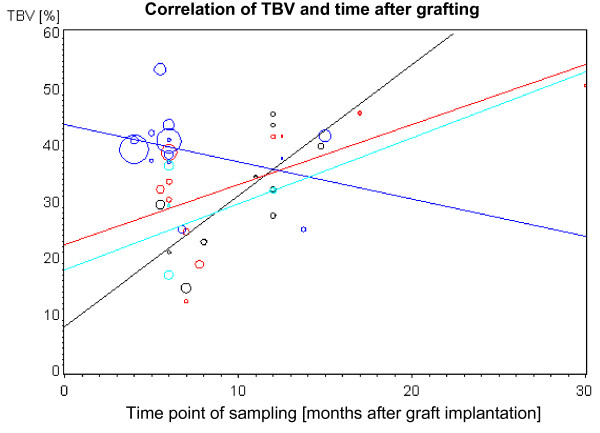
**Correlation of TBV and time after grafting**. The size of the bubbles reflect the relative weight of the value. Black: Bio Oss^®^, red: Bio Oss^® ^with autogenous bone, blue: autogeneous bone, green: β-TCP.

The weighted regression of TBV against the time point of sampling shows the development of the bone volume during time (Fig. 1). Interestingly, while Bio Oss^®^, Bio Oss^® ^with autogenous bone and β-TCP show a steep increase the TBV of autogenous bone (without any additional grafting material) is decreasing. The increase of TBV during time in the Bio Oss^® ^group can be considered as statistically significant.

**Figure 2:**
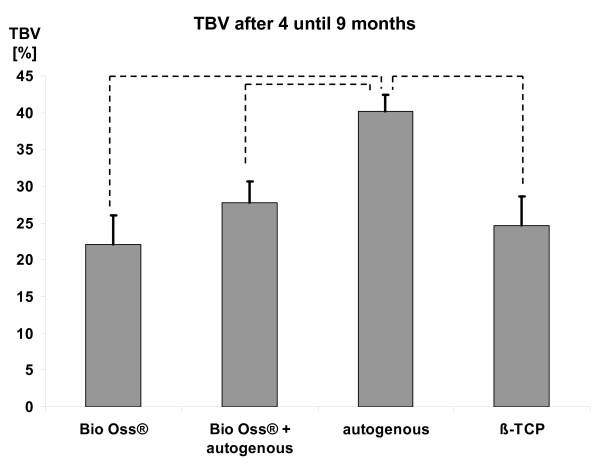
**TBV after 4 until 9 months**. Shown are the values and the SD of the weighted mean. The dashed lines mark the statistical significant differences.

**Figure 3:**
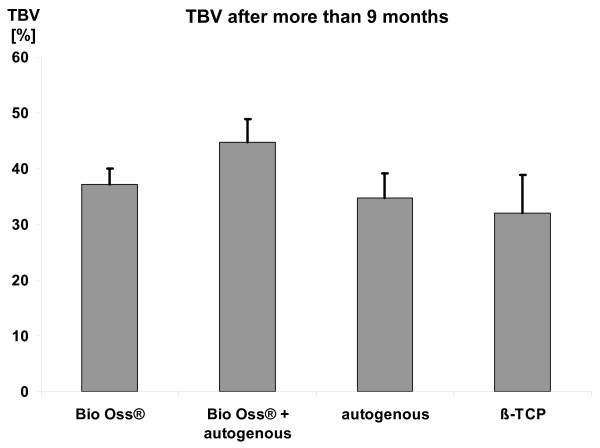
**TBV after more than 9 months**. Shown are the values and the SD of the weighted mean.

Regarding Fig. 1 it is striking that there are two clusters of sampling. The first cluster comprise four until nine months after initial surgery and the second cluster span the time from nine months onwards. To compare the TBV depending on the grafting material the mean values were calculated for these two clusters. Because it is reasonable to weight the study results regarding the number of observations and the standard deviation both the "normal" and the adjusted mean values were calculated. In a relatively early phase after implantation the autogenous bone shows the highest TBV values. This was statistically significant (Fig. 2). Interestingly, the different TBV levels approximate during the time. After 9 months no statistically significant differences can be detected between the various grafting materials (Fig. 3). However, there was a strong tendency that Bio Oss^® ^with autogenous bone shows the highest TBV values.

## Discussion

External sinus floor augmentation has proven to be very effective in increasing bone volume in edentulous maxillary areas. Due to the significant resorption in the posterior maxilla following teeth extraction [42] there is often not enough bone volume to ensure the stability of dental implants [43]. Elevation and augmentation of the maxillary sinus can increase the bone height in the posterior area of the maxilla [1,2]. Autogenous bone grafts obtained from the patient himself is very successful in bone regeneration and serves as a gold standard [10]. However, the explant of autogeneous bone requires an extra donor site surgery and is associated with an extra risks for morbidity and complaints, particularly when bone from the iliac crest is harvested [11]. Various bone grafting materials have been used as alternatives or supplements to autogenous bone such as demineralized freeze-dried bone (DFDBA), hydroxyapatite, β-tricalcium phosphate (β-TCP), anorganic deproteinized bovine bone [8] or combination of these materials. Bone grafting materials may produce bone formation by osteogenesis, osteoinduction or osteoconduction. Whereas osteogenesis is obtained by providing osteogenic cells and matrix directly in the graft (e.g. autogenous bone, distraction osteogenesis [44]), osteoinduction postulates that the grafted material is chemotactic to undifferentiated progenitor cells inducing them to differentiate into osteoblasts [31,45]. Osteoconduction permits outgrowth of osteogenic cells from existing bone surfaces into the graft material [31]. Although these mechanisms have been described in detail, the question remains which bone grafting material is most suitable in external sinus floor augmentation at the histological level.

One important finding of this study is that there is only little evidence for most of the grafting materials. Only anorganic deproteinized bovine bone (Bio Oss^®^) and pure-phase β-TCP (in most cases Cerasorb^®^, Curasan Pharma GmbH, Kleinostheim, Germany, was used) as well as autogenous bone (and combinations of these materials) were found to present evaluable data for meta-analysis. Interestingly, no reports regarding allergic reaction or infections caused by implantation of grafting material were described in the articles.

With regard to the TBV autogenous bone reaches the highest values during the first 9 months. This difference to the other materials was statistically significant. That's means that the percentage of mineralized bone was the highest. That is not surprisingly, because in the specimens of the other groups there are of course particles of the heterologous or alloplastic grafting material diminishing the percentage of the bone. Logically consistent the TBV shows the lowest values in the Bio Oss^® ^and β-TCP groups. In contrast to this early phase there is no statistically significant difference between the grafting materials in the later phase anymore. Moreover, the values of the Bio Oss^® ^group and Bio Oss^® ^with autogenous bone show higher mean values than the pure autogenous bone, whereas the mean value of β-TCP is almost equal to autogenous bone. There could be two adverse effects after the initial grafting procedure. On the one hand bone is resorbed because in no case was any functional load on the grafting material (The samples of the patients were taken before the implant was in function). On the other hand the TBV in the Bio Oss^® ^and β-TCP groups increased during time. That means that the grafting material is at least partially resorbed and replaced by bone. (Reduction of soft tissue volume hardly produce an increase of TBV because in sinus lift procedures soft tissue is very rare in the grafted material.) The first effect is well known and occurs in the alveolar bone usually after tooth extraction when the functional load is reduced or absent [42]. Additionally, there are reports in literature that up to 55% of the augmented autogenous bone resorbs during the first 6 months [46,47]. The second effect reflects osteoinductive or at least osteoconductive properties of the non-autogenous grafting materials. Tadjoedin and colleagues describe in pure Bio Oss^® ^grafts, that bone growth takes place through the guidance of osteogenic cells from existing bone surfaces of the grafted particles. This leads to the formation of woven bone between the grafted particles connecting them together into a mass of mineralized tissue [31]. When autogenous bone is mixed with Bio Oss^® ^the human bone particles act as a source of bone cells [48,49] providing more osteogenic cells and thus accelerating new bone formation. This is in line with an former study reporting that bone formation in a patient was faster in a mixed graft of Bio Oss^® ^and autogenous bone than in a graft of Bio Oss^® ^alone [50]. Bio Oss^® ^seem to prevent bone loss and increase new bone formation but it is unclear wether or how fast the Bio Oss^® ^particles will be resorbed. Both no resorption after six years [51] and slow resorption [31] are reported in literature.

In contrast to Bio Oss^® ^there are reports that β-TCP is fully resorbed in 12 to 18 months and is replaced by bone that is similar both functionally and anatomically to the original bone [30]. Regarding the TBV there no statistically significant differences between Bio Oss^® ^and β-TCP although the combination of Bio Oss^® ^with autogenous bone shows the highest value in the later phase. Because β-TCP was used as a grafting material only without bone in the evaluated studies it might be that an additional supplement of autogeneous bone could increase the TBV too. The mechanism of preventing fast resorption and of increasing the TBV after about one year is probably very similar to Bio Oss^®^.

## Conclusion

Taken together, comparability of Bio Oss^® ^with or without autogenous bone and β-TCP to autogenous bone for sinus grafting can be regarded as evidence based concerning the histological bone structure after about 9 months. However, the augmented material contain more mineralized bone tissue 4–9 months after grafting when only autogenous bone is used. From a clinical point of view, the use of autogenous bone is advantageous if a prosthetic rehabilitation (with functional loading) is expected within 9 months. In other cases the use of Bio Oss^® ^in combination with autogenous bone seems to be preferable. Donor side morbidity is ignored in this conclusion.

When reviewing the literature and doing a meta-analysis there is one additional thing you have to bear in mind: the publication bias. That means that most of all authors report only from good results especially in case reports or case series. Bad or unwanted results are often neglected and not published in international journals. Therefore, even the results of this meta-analysis – although representing the highest grade of evidence – show presumably slightly to optimistic numbers.

## Competing interests

The authors declare that they have no competing interests.

## Authors' contributions

JH conceived the study and drafted the manuscript. MS carried out the literature research. RD and CN calculated the statistics. MO, NK and UM participated in its design and coordination and helped to draft the manuscript. All authors read and approved the final manuscript.
